# A Theoretical Study on the Phosgenation of 2,4-Toluenediamine (2,4-TDA)

**DOI:** 10.3390/polym14112254

**Published:** 2022-05-31

**Authors:** Ravikumar Thangaraj, Tamás Horváth, R. Zsanett Boros, Béla Viskolcz, Milán Szőri

**Affiliations:** 1 Institute of Chemistry, University of Miskolc, Miskolc-Egyetemváros A/2, H-3515 Miskolc, Hungary; ravikumar8019@gmail.com (R.T.); horvathtamas010107@gmail.com (T.H.); bela.viskolcz@uni-miskolc.hu (B.V.); 2 Higher Education and Industrial Cooperation Centre, University of Miskolc, H-3515 Miskolc, Hungary; 3 BorsodChem Zrt, Bolyai tér 1., H-3700 Kazincbarcika, Hungary; creamy0711@gmail.com

**Keywords:** phosgenation, toluenediamine, toluene diisocyanate, G3MP2B3, carbamic chloride

## Abstract

Industrially relevant phosgenation mechanisms of 2,4-toluenediamine (2,4-TDA) were investigated using G3MP2B3 model chemistry. Six reaction pathways had been explored, which resulted in the formation of toluene diisocyanate (2,4-TDI) including different scenarios of the ‘phosgenations first’ and ‘consecutive phosgenations’ mechanisms in both gas and condensed phases. Two possible ‘phosgenations first’ mechanisms show superior to the others in terms of energy, regardless of which phases are considered. Due to the o-dichlorobenzene (ODCB) solvation, the reaction barriers are dramatically reduced compared to the gas-phase reaction mechanism and the solvent effect can be described by linear relationship. Standard enthalpy of formation value was also recommended for 2,4-TDA (59.3 kJ/mol) and 2,4-TDI (−94.1 kJ/mol), as well as for the gas-phase intermediates (IM).

## 1. Introduction

Toluene diisocyanate (TDI) is an organic compound, which is a colorless to pale yellowish liquid with a sharp pungent odor used predominantly in the production of polyurethanes (primarily flexible foams) and consumer products, such as coatings, elastomers, adhesives, and sealants [1]. It is also used as curing agent in solid propellant formulations [2]. It is insoluble in water and miscible in most common organic solvents [3]. The most important isomers of TDI are toluene-2,4-diisocyanate (2,4-TDI) and toluene-2,6-diisocyanate (2,6-TDI), commercially produced in different weight ratios. The most common ratio is an 80/20 mixture of 2,4-TDI and 2,6-TDI [3]. The global demand for TDI was 2.37 million tons in 2020, and it is forecasted to increase to about 2.77 million tons by end of 2022 [4].

**Figure 1:**
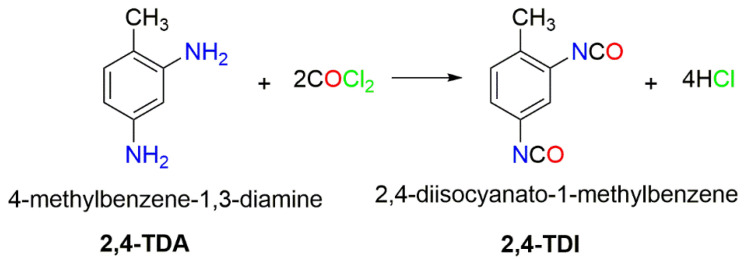
Reaction scheme for the phosgenation of TDA.

As shown in Figure 1, the 2,4-TDI is primarily produced by the reaction of toluenediamine (4-methylbenzene-1,3-diamine, abbreviated as 2,4-TDA) with phosgene (COCl_2_) in a continuous reactor, and the resultant crude 2,4-TDI is purified by distillation. The distillation residue formed is hydrolyzed at <230 °C under absolute pressures of less than 30 bar [5]. For these process, the reactive phosgene compound is produced by passing purified carbon monoxide and chlorine gas through a bed of porous activated carbon [6,7].

The industrial phosgenation of TDA is carried out in a chemically inert organic solvent, maintaining a suitable temperature from 40 to 150 °C [8]. The gas-phase phosgenation of TDA also can be carried out in the presence of inert gas or vapors of an inert solvent and subsequently heated to a temperature of 300–400 °C [9]. However, the reaction of amine and phosgene in the liquid phase is very rapid at all industrial temperatures and pressures, only a good mixing is desired to suppress the secondary reactions [10]. Organic solvents such as benzene, toluene, xylene, chlorinated aromatic hydrocarbons, and petroleum hydrocarbons, which are inert with hydrogen chloride, phosgene, and isocyanate can be employed [11]. While using ortho-dichlorobenzene (ODCB) as a solvent, mild operating conditions (31.4–100 °C) [12] can be applied, which are mainly due to the high boiling point of ODCB (T_b_ = 180.5 °C). The unreacted phosgene and hydrochloric acid are recycled and utilized in the technology of TDI production [13]. Performing the phosgenation reaction in the gas phase is more economical and environmentally benign than in the liquid phase due to low residence time, and reduced usage of solvent and energy [14]. However, there are side reactions in the gas phase because of the evaporation of amines leading to the formation of deposits with unresolved structures in the reactors [15]. Hence, a thorough understanding of the fundamental reactions is necessary. In general, the phosgenation of diamines [15] takes place by two reaction mechanisms: (i) the transformation of both amino groups into carbamoyl chloride followed by the two consecutive HCl elimination (termed as “phosgenations first”), and (ii) one of the amine groups turns into isocyanate while the other amine group remains intact and the same occurs with the other amine group (termed as “stepwise phosgenations”).

On one hand, there is an increasing request to understand industrial processes at the molecular level in order to use this information for the further process development. Many theoretical investigations are going on in the field of urethane industry, including phosgene production in presence of [NEt_3_Me]Cl/[NEt_3_Me][Cl_3_] catalyst system [16], thermal decomposition of phosgene [17], the formation of other diamine intermediate (MDA), the phosgenation of MDA [15], the liquid structure of different isocyanates [18,19], urethane formation with an excess of isocyanate or alcohol [20], and in presence of catalysts [21], just to name a few. On the other hand, the chemical industries largely use the phosgenation of 2,4-TDA. Its reaction mechanism and the corresponding energy profile are less explored. To the best of our knowledge, the thermochemical properties of 2,4-TDI and their intermediates have not yet been reported. Therefore, the current work aims to investigate the reaction mechanism of the phosgenation of 2,4-TDA using quantum chemical calculations. The thermodynamic profiles of the phosgenation reaction of 2,4-TDA in the solvent ODCB and their gas-phase reaction were compared. Moreover, the effect of temperature on the thermodynamic properties of gas-phase and liquid-phase reactions for the formation of 2,4-TDI was studied.

## 2. Computational Methods

G3MP2B3 model chemistry [22] is considered a reasonable compromise between the accuracy and the computational costs of the system of interest; therefore, G3MP2B3 was applied to compute thermochemical properties of the species involved in the title reaction, as implemented in the Gaussian09 program package [23]. As part of this protocol, geometry optimizations were carried out using the B3LYP/6-31G(d) level of theory [24] by applying the “tight” convergence criterion. Normal mode analysis was performed on the optimized structures at the same level of theory to characterize the identities of the computed species on the potential energy surface (PES). Furthermore, the transition-state (TS) structures also were confirmed to locate first-order saddle points on the PES by visual inspection of the intramolecular motions corresponding to the imaginary wavenumber using GaussView 6 [25]. Intrinsic reaction coordinate (IRC) calculations [26] were carried out to map the minimal energy pathways (MEP). As a first proxy, the zero-point corrected relative B3LYP/6-31G(d) energy (Δ*E*_0,B3LYP/6-31G(d)_) had been calculated. To refine the electronic energy, additional single point calculations of the critical points of the PES were carried out using QCISD(T)/6-31G(d) (including MP2/6-31G(d) level of theory) and MP2/GTMP2 levels of theories based on B3LYP/6-31G(d) geometries according to the G3MP2B3 composite method. Harmonic wavenumbers obtained at B3LYP/6-31G(d) level of theory were scaled by a factor of 0.96 [22] for refining the accuracy of the thermodynamic properties, such as zero-point corrected relative energy (Δ*E*_0_,_G3MP2B3_), relative enthalpy (Δ*H*°_G3MP2B3_), the standard enthalpy of formation (Δ_f,298.15K_*H*°(g)), relative Gibbs free energy (Δ*G*°_G3MP2B3_), and entropy (*S*°) under standard condition (T = 298.15K and P = 1 atm). The Δ_f,298.15K_*H*°(g) were obtained by group additivity (GA) role using online NIST tool [27], as well as by atomization scheme (AS) using the standard enthalpy of formation for atoms from CCCBDB database [28].

The integral equation formalism of the polarizable continuum model (PCM) with the radii and non-electrostatic terms of Truhlar and co-workers (SMD) [29] was used to mimic implicitly the surrounding ortho-dichloro-benzene (ODCB) environment of the phosgenation. The SMD model had been applied for the re-optimization of all the gas-phase structures meaning the SMD treatment of ODCB was fully integrated into the G3MP2B3 protocol.

## 3. Results and Discussion

### 3.1. Gas-Phase Reaction Mechanism

**Figure 2:**
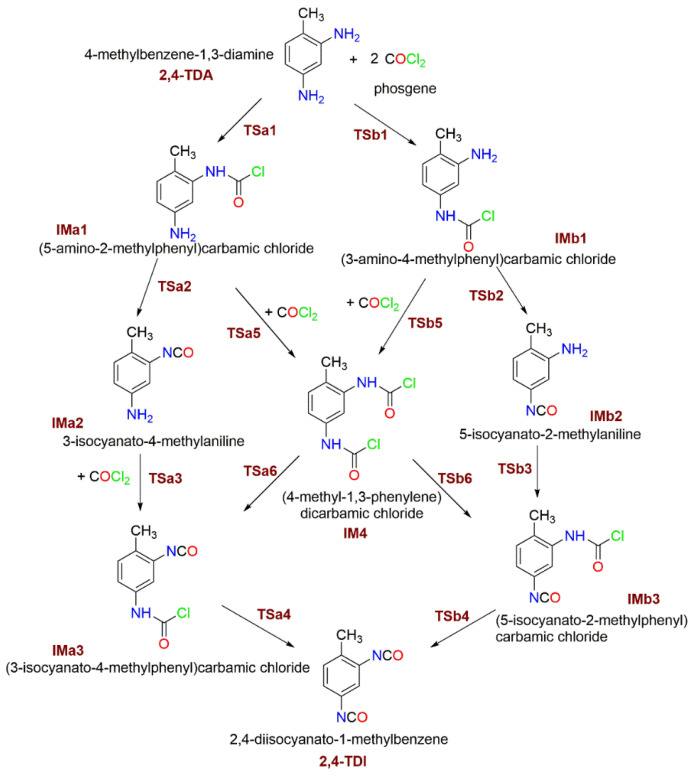
Studied reaction mechanism of the 2,4-TDA phosgenation.

**Figure 3:**
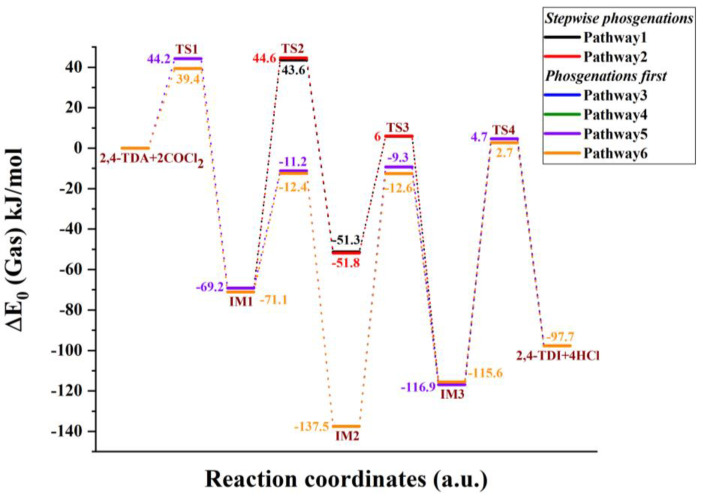
G3MP2B3 energy profile (zero-point corrected) for the phosgenation of 2,4-TDA in the gas phase.

**Figure 4:**
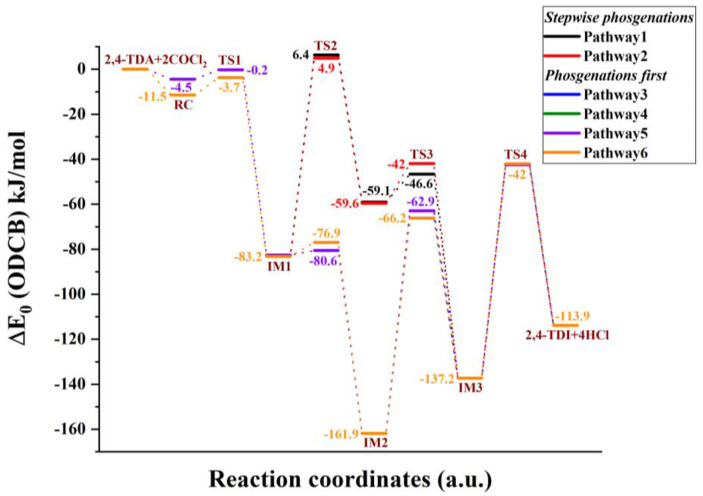
G3MP2B3 energy profile (zero-point corrected) for the phosgenation of 2,4-TDA in ortho-dichlorobenzene (ODCB).

Due to the asymmetric arrangement of the amine group in 2,4-TDA, six different phosgenation reaction pathways can be considered and they can be classified into two types, such as “stepwise phosgenations” and “phosgenations first”, as shown in Figure 2. The “phosgenations first” mechanism means that both amines transform to carbamic chloride groups in a consecutive manner and then HCl-elimination takes place to give the isocyanate groups. In the “stepwise phosgenations” mechanism, one of the amine groups turns into isocyanate while the other amine group remains intact, and the same occurs with the other amine group. Each type has different reaction pathways, “stepwise phosgenations” have two reaction pathways (noted as Pathway 1 and Pathway 2 in Figure 3 and Figure 4), and “phosgenations first” have four reaction pathways (noted as Pathway 3–6 in Figure 3 and Figure 4).

**Figure 5:**
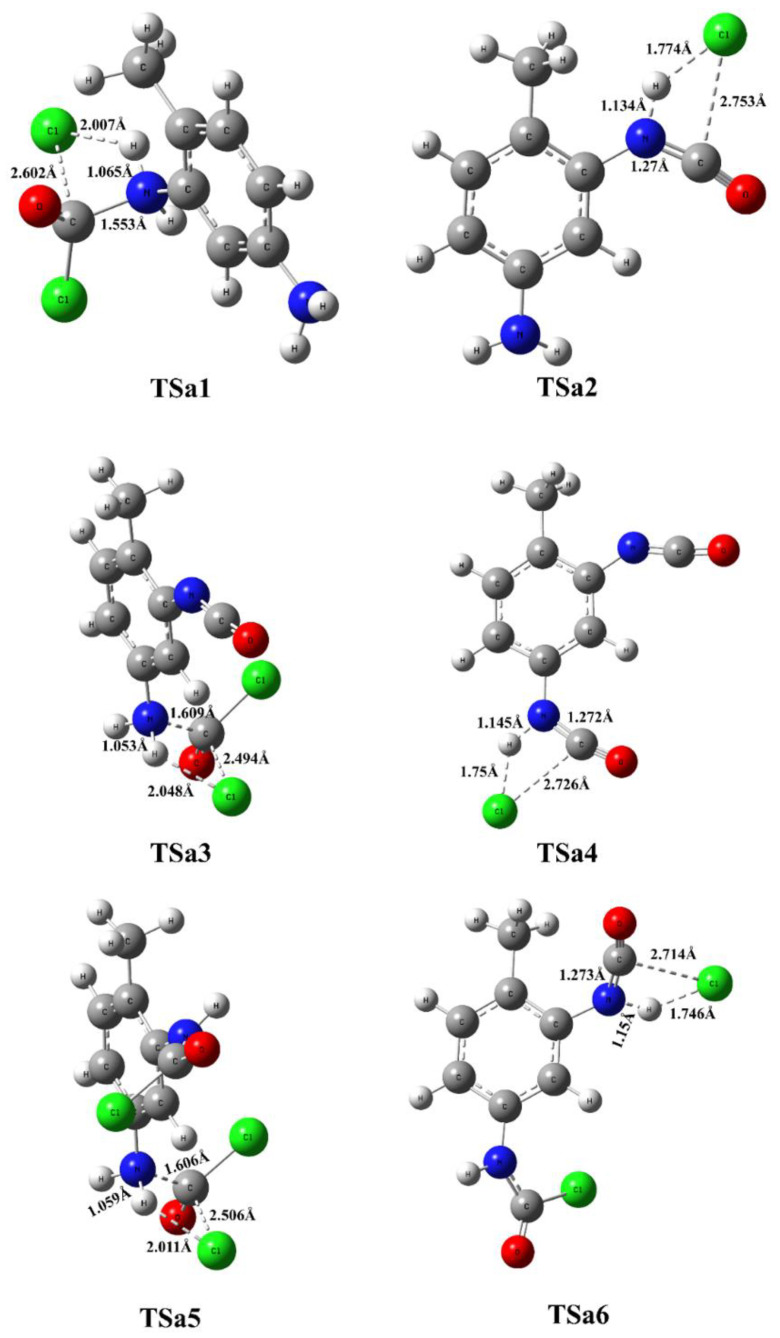
Transition-state structures for the phosgenation of 2,4-TDA in gas phase.

**Figure 6:**
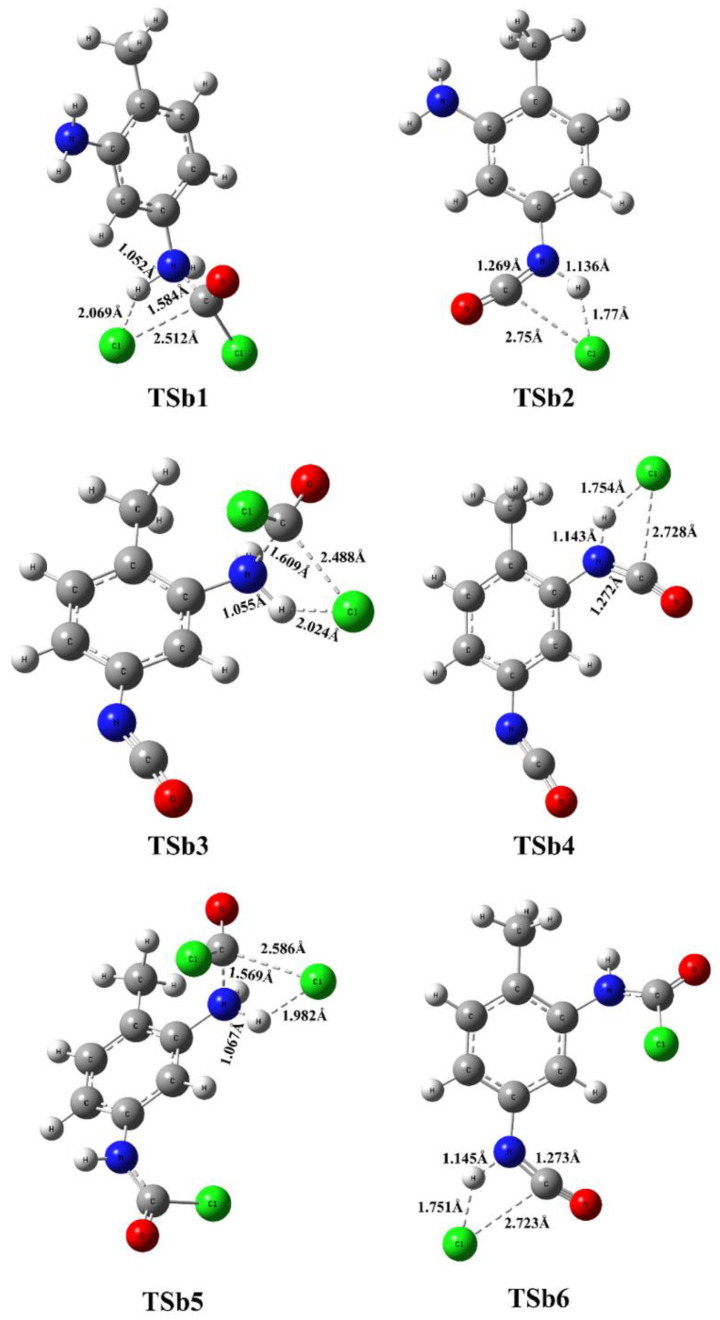
Transition state structures for the phosgenation of 2,4-TDA in gas phase.

In the first elementary step, one of the amine groups turns into carbamic chloride via a four-membered transition state, in which synchronous phosgene addition and HCl elimination take place. If the reaction center is close to the methyl group, that is TSa1, the reaction barrier is roughly 5 kJ/mol higher (Δ*E*_0_ = 44.2 kJ/mol) than in the case of amine in position 3 (TSb1, Δ*E*_0_ = 39.4 kJ/mol), as shown in Figure 3, for the gas-phase reaction (as discussed later, the potential energy surface for the ODCB phase reaction shows the same trends shown in Figure 4), and in Table 1. This is also manifested in the transition-state structure in such a way that the critical bond distance for the C-Cl bond being broken is significantly larger in the case of Tsa1 (2.602 Å) than for TSb1 (2.512 Å) and the released chlorine approaches the hydrogen amine closer (2.007 Å vs. 2.069 Å), as shown in Figure 5 and Figure 6. Both transition states correlate with exothermic products, namely (5-amino-2-methylphenyl)carbamic chloride (IMa1, Δ_r_*H*^0^ = −65.5 kJ/mol) and (3-amino-4-methylphenyl)carbamic chloride (Imb1, Δ_r_*H*^0^ = −67.8 kJ/mol).

In the case of “stepwise phosgenations”, the carbamic chloride intermediate IMa1 or Imb1 undergoes HCl-elimination resulting in the formation of the first isocyanato group, namely 3-isocyanato-4-methylaniline (IMa2) and 5-isocyanato-methylaniline (Imb2), respectively. Their relative energies (IMa2 (Δ*E*_0_ = −51.3 kJ/mol), Imb2 (Δ*E*_0_ = −51.8 kJ/mol)) are higher than the intermediate of the previous step (IMa1 (Δ*E*_0_ = −69.2 kJ/mol), Imb1 (Δ*E*_0_ = −71.1 kJ/mol)). Therefore, the corresponding transition states (Tsa2 and TSb2) lie high (Δ*E*_0_ = 43.6 kJ/mol for Tsa2 and Δ*E*_0_ = 44.6 kJ/mol for TSb2), which can be understood by looking at the drastically elongated C-Cl bond in the transition-state structures in Figure 5 (2.753 Å for Tsa2) and Figure 6 (2.750 Å for TSb2). This HCl elimination step is the most energy-consuming one in the “stepwise phosgenations” mechanism. Considering the further step either from IMa2 (→ Tsa3 → IMa3 → 2,4-TDI) or Imb2 (→ TSb3 → Imb3 → 2,4-TDI), it turned out that the transformation of the second amine group into isocyanate showed similar energy profile as the first one.

For instance, IMa2 reacts with excess phosgene and forms carbamic chloride compound (IMa3, 3-isocyanato-4-methylphenyl)carbamic chloride) and the exoergicity of this elementary step is −65.6 kJ/mol, which is only slightly different from that of IMa1 (Δ*E*_0_ = −69.2 kJ/mol). However, the Imb3 pathway is less exoergic by 7.4 kJ/mol (Δ*E*_0_ = −63.7 kJ/mol vs. Δ*E*_0_ = −71.1 kJ/mol for Imb1). Therefore, one can state that the reactivity of these amine groups only slightly interferes, which is also confirmed by the high similarity between corresponding transition states and intermediate structures (therefore these structures are not discussed here thoroughly). Regardless of the small deviations, the energy pattern for an isocyanate group formation with the “stepwise phosgenations” mechanism can be described as follows: the first step is exothermic with a transition state lying below the transition state of the endothermic second step. Due to the high exothermicity of the synchronous phosgene addition and HCl-elimination step the overall reaction is strongly exothermic with the formation of the TDI product and four HCl molecules (Δ*H*^0^ = −81.4 kJ/mol).

Regardless of IMa1 or Imb1 formed in the first reaction step of the “phosgenations first”, mechanism can take place via Tsa5 or TSb5 transition state, respectively. The resulted intermediate is the same, that is 4-methyl-1,3-phenylene dicarbamic chloride (IM4) and HCl. The relative energies of Tsa5 and TSb5 are close to each other (−11.2 kJ/mol for Tsa5 and −12.4 kJ/mol for TSb5), and both are below the entrance level even in the case of gas-phase reactions. They are also similar in structure to each other, as well as Tsa1, Tsa3, TSb1, and TSb3. The largest variation in the parameters can be found in the case of the C-Cl distance (a bond being broken), which is the smallest for TSb3 (2.488 Å) and the largest for Tsa1 (2.602 Å).

IM4 with the two carbamic chloride groups can turn into either IMa3 or Imb3, regardless of forming through either Tsa2 or TSb2, making four possible pathways for the “phosgenations first” mechanism. These HCl-eliminations (Tsa6 and TSb6) are structurally similar to the transition states of final HCl-eliminations (Tsa4 and TSb4); therefore, they are all discussed at once. The C-Cl distance is elongated (2.723–2.753 Å) significantly, which means that the C-Cl is already partially being broken and this liberated chlorine atom synchronously approaches the amine hydrogen in the distance of 1.751–1.774 Å in a planar four-centered transition state. These HCl-elimination reactions are all endoergic by 17.8 kJ/mol and 19.2 kJ/mol.

Comparing the two types of reaction mechanisms from an enthalpic perspective, “stepwise phosgenations” have an exothermic and then an endothermic step in consecutive manner, whereas the “phosgenations first” type has two exothermic steps at the beginning and two endothermic steps at the end.

### 3.2. Thermochemical Properties of the Intermediates

The thermodynamic properties also can be calculated for all the intermediates, reactant and product in the gas phase at thermodynamic standard conditions and all of them are tabulated in Table 2. 

Standard molar enthalpy of formation (Δ_f,298.15K_*H*^0^(g)) was directly derived from our G3MP2B3 results for the atomization scheme (AS), calculated by using the accurate atomization enthalpy values (Δ_atom_H^0^(^3^C) = 716.68 ± 0.45 kJ/mol, Δ_atom_H^0^(^2^H) = 218.00 ± 0.01 kJ/mol, Δ_atom_H^0^(^4^N) = 472.68 ± 0.40 kJ/mol, Δ_atom_H^0^(^3^O) = 249.23 ± 0.00 kJ/mol, Δ_atom_H^0^(^2^Cl)= 121.31 ± 0.01 kJ/mol) from CCBDB database and the reaction enthalpy of the following hypothetical reaction with the stochiometric numbers x, y, z, q and w:

x^3^C + y^2^H + z^4^N + q^3^O + w^2^Cl = ^1^C_x_H_y_N_z_O_q_Cl_w_

However, the NIST group additivity tool (GA) was used to generate values for TDA and TDI. In the GA model, the group increment for methyl group was −42.7 kJ/mol, while that of the phenyl ring is 0 kJ/mol. The enthalpy contribution from the amine group bonded to aromatic carbon (C_a-ring_) is 20 kJ/mol. Finally, enthalpy increments of C_a-ring_-C, C_a-ring_-H and C_a-ring_-N were 23 kJ/mol, 14 kJ/mol and −2 kJ/mol, respectively. All these additivity values were obtained from the NIST tool [27]. In case of TDI, the aromatic isocyanate group increment (−61.2 kJ/mol) was adopted from our previous work [15,30]. In both cases, the AS and GA values are within 3 kJ/mol, which also suggest that the energetics of the title reaction obtained with G3MP2B3 are consistent with the literature. Surprisingly, there is no reported standard enthalpy of formation for any of the compounds listed in Table 2 to the best of our knowledge. As also can be seen in Table 2, only TDA has endothermic formation and formation of IMa2 and IMb2 have slightly exothermic while the remaining intermediates (IMa1, IMb1, IMa3, IMb1, IM4) and TDI are all highly exothermic product of the phosgenation. However, the overall reaction of the 2,4-TDI formation is exothermic, 2,4-TDI is reactive enough to form highly thermally and mechanically stable frameworks, such as isocyanate dimers and isocyanurates [31], but such condensed phase reactions is out of the scope of the current study. For completeness, the entropy content (*S*°(g)) and heat capacity values (***C_v_***(g)) are also tabulated in Table 2 for all species studied.

### 3.3. Effect of Solvent and Temperature

As shown earlier, the first activation barrier of gas-phase phosgenation (both TSa1 and TSb1) lies relatively high, and this is a crucial step in this industrially important system. To reduce the activation energy, ODCB is used in the chemical industry as an inert, but polar solvent (the permanent electric dipole moment of ODCB is 2.27 D). Furthermore, to get closer to the industrial relevant condition, two different temperature effects were studied (273.15 K, and 423.15 K) and their thermochemical properties are shown in Table 1. Due to the polar environment provided by ODCB, the relative energies of the transition states and intermediates are all drastically reduced. Relative energies of the first transition states in both directions become submerged (Δ*E*_0_ = −0.2 kJ/mol for TSa1 and Δ*E*_0_ = −3.7 kJ/mol for TSb1) and are kinetically easily accessible.

**Figure 7:**
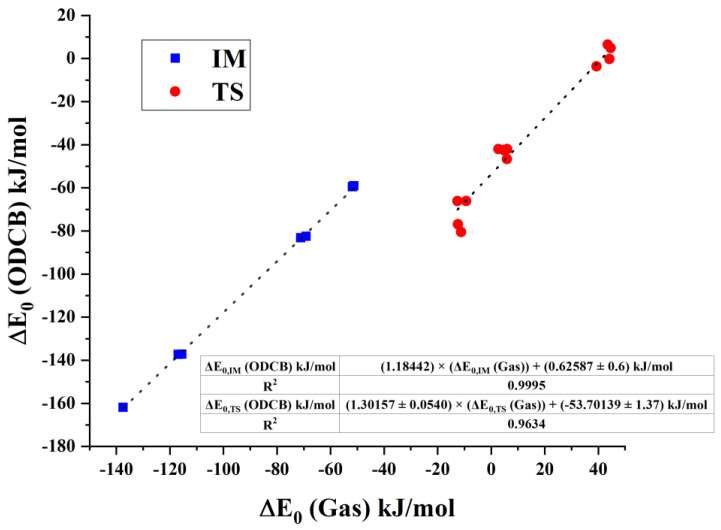
Comparison of intermediate and transition-state energies between gas phase and ODCB obtained at G3MP2B3. Fitted plots are marked in dotted lines.

Comparing Figure 3 and Figure 4, the potential energy surfaces in gas phase and ODCB remained similar, therefore the relative energies of gas phase against those in ODCB are plotted in Figure 7, and they show a very strong linear relationship with the R^2^ value of 0.9634, and 0.9995 for the transition-state (TS) and the intermediate (IM) structures, respectively. Analyzing the fitted expression for TS relative energies, the gas-phase relative energies are all multiplied by a factor of 1.3. Then, this effect is overcompensated for the positive relative energy state by a general strong shift (−53.7 kJ/mol) toward smaller energies as the intercept indicates. Since all intermediates are below the energy level of the reactants, the 1.18 slope value resulted in a significant decrease in their energies.

The relative enthalpies are not significantly affected by changing the temperature from 298.15 K to 423.15 K. The largest absolute deviation was 2.6 kJ/mol, regardless whether gas-phase or ODCB phase values were considered.

### 3.4. Comparison of the Relative Energies from G3MP2B3 and B3LYP/6-31G(d)

**Figure 8:**
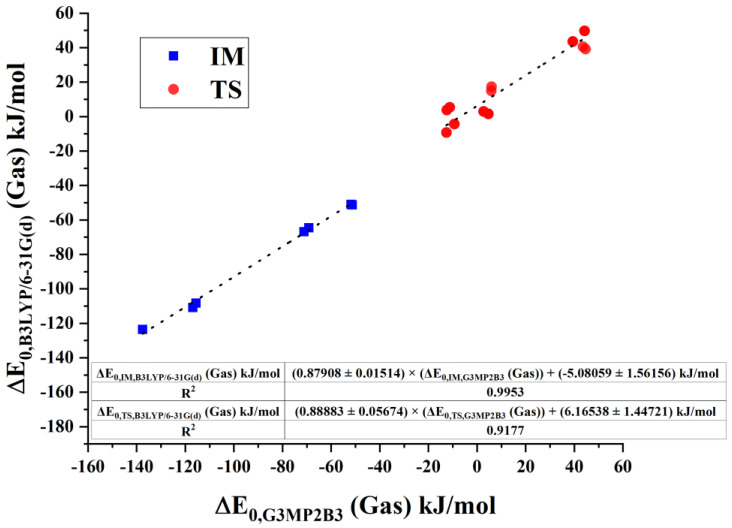
Comparison of intermediate and transition state energies in gas phase obtained by B3LYP/6-31G(d) level of theory and G3MP2B3. Fitted plots are marked in dotted lines.

**Figure 9:**
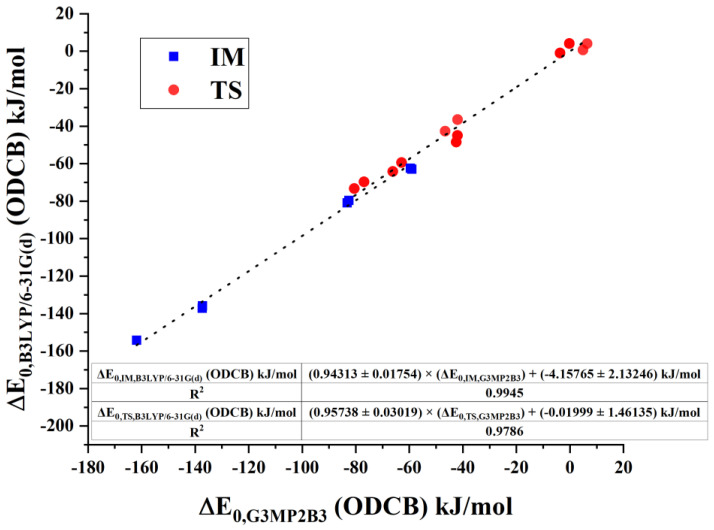
Comparison of intermediate and transition-state energies in ODCB obtained by B3LYP/6-31G(d) level of theory and G3MP2B3. Fitted plots are marked in dotted lines.

Phosgenation of amines can be studied for larger analogous systems, although the G3MP2B3 computation is not suitable for a larger system. To provide a trade-off alternative according to accuracy and computation time, we studied the correlation between the G3MP2B3 and B3LYP/6-31G(d) relative energies. As shown in Figure 8 for gas-phase energies and in Figure 9 for the ODCB phase, G3MP2B3 and B3LYP/6-31G(d) energies are in a strong linear relationship for transition states (TS), and intermediates (IM) with the R^2^ value of 0.9177, and 0.9953, respectively, for gas phase and 0.9786, and 0.9945, respectively, for ODCB. For both surrounding media, slope values for TS and IM agree with the uncertainty of the fit, but they differ according to gas-phase (roughly 0.88) or ODCB (roughly 0.95) results. The magnitude of the intercepts provides a relatively small shift in the relative-energy values, from 0.02 kJ/mol to 6.2 kJ/mol. Their deviations for IM and TS are close to or somewhat above their uncertainties.

### 3.5. Thermochemistry of the Studied Reaction at Different Conditions

The phosgenation of 2,4-TDA in the gas-phase and ODCB environments was studied at two different temperatures, 298.15 K and 423.15 K, to explore the temperature dependence on the thermodynamics of the studied reaction mechanism. Relative enthalpies and Gibbs free energies obtained at the mentioned temperature and 1 atm pressure are given in Table 1. 

No significant change in the relative enthalpies (Δ*H*_G3MP2B3_(T)) (0.0 and 2.6 kJ/mol for gas phase and −0.2 and 2.6 kJ/mol for ODCB) was observed in both reaction environments while increasing the temperature from 298.15 K to 423.15 K. However, the relative Gibbs free energies (Δ*G*^0^) were substantially increased from −33.5 to 22.9 kJ/mol for gas phase and −32.3 to 24.2 kJ/mol for ODCB. 

## 4. Conclusions

In this work, the energy profile of the six different possible reaction mechanisms for the 2,4-TDA phosgenation were discussed based on the results obtained by G3MP2B3 composite method and B3LYP/6-31G(d) level of theory, which can be classified as follows:

(a) “Phosgenations first”: first amine groups turn into carbamic chloride groups and then the isocyanate groups form;
(b) “Stepwise phosgenations”: amine group presented in ortho, or para, position converts as a carbamic chloride and then it loses HCl and forms isocyanate. The same step repeats for the other position and forms another isocyanate group.

Our key findings of these reaction mechanisms are the followings:

1. The addition of the first COCl_2_ is the rate limiting step for the reaction, regardless of which mechanism is taking place;
2. The activation barriers for the gas-phase reaction are relatively high and the ODCB has reduced the activation barriers for all the reaction pathways;
3. For transition states and intermediates, the gas phase and ODCB phase relative energies show strong linear correlation, and the presence of ODCB medium causes drastic shrinking in energy level of the transition state as one compares them with the gas-phase values. For the intermediates, the relative energies lowered by a factor of 1.18;
4. Comparing the energy profile of the “phosgenations first” and “stepwise phosgenations”, the former has two exothermic consecutive elementary steps and then two consecutive endothermic ones, while the latter has consecutive exothermic and endothermic steps. Therefore, the “phosgenation first” seems to be the dominant channel due to both thermodynamic and kinetic points of view;
5. Standard enthalpy of formation value is recommended for 2,4-TDA (59.3 kJ/mol) and 2,4-TDI (−94.1 kJ/mol), as well as for the gas-phase intermediates (IM);
6. Relative energies obtained by G3MP2B3 and B3LYP/6-31G(d) has linear relation in both the gas phase and ODCB for transition states and intermediates. Therefore, B3LYP/6-31G(d) computation can be an excellent compromise between accuracy and computation time for phosgenation reactions.

## Figures and Tables

**Table 1:** G3MP2B3 thermochemical properties calculated in the gas phase and ortho-dichlorobenzene (ODCB), including zero-point corrected relative energies (Δ*E*_0,G3MP2B3_), relative enthalpies (Δ*H*^0^_G3MP2B3_), and relative Gibbs free energies (Δ*G*^0^_G3MP2B3_).

	Δ*E*_0,G3MP2B3_		Δ*H*°_G3MP2B3_				Δ*G*°_G3MP2B3_			
Solvent	-	ODCB	-	ODCB	-	ODCB	-	ODCB	-	ODCB
T in K	0		298.15		423.15		298.15		423.15	
TDA + 2COCl_2_	0.0	0.0	0.0	0.0	0.0	0.0	0.0	0.0	0.0	0.0
RC1	0.0	−4.5	0.0	−4.7	0.0	−3.8	0.0	48.1	0.0	70.0
RC2	0.0	−11.5	0.0	−11.1	0.0	−10.0	0.0	38.4	0.0	58.9
Tsa1	44.2	−0.2	43.3	−1.8	43.4	−2.0	92.7	52.0	113.4	74.8
IMa1 + HCl	−69.2	−82.5	−65.5	−78.8	−64.5	−77.8	−61.2	−73.0	−59.6	−70.8
Tsa2 + HCl	43.6	6.4	48.1	10.2	48.9	10.8	48.4	15.9	48.4	18.0
IMa2 + 2HCl	−51.3	−59.1	−42.8	−50.6	−41.4	−49.3	−83.5	−89.1	−100.8	−105.5
Tsa3 + 2HCl	5.9	−46.6	12.8	−39.6	14.0	−38.4	24.6	−24.6	29.3	−18.6
IMa3 + 3HCl	−116.9	−137.3	−105.2	−125.6	−103.0	−123.4	−140.1	−156.4	−155.2	−169.8
Tsa4 + 3HCl	4.7	−42.5	17.3	−30.3	19.4	−28.4	−21.1	−62.5	−37.7	−77.6
Tsa5 + HCl	−11.2	−80.6	−8.7	−77.1	−7.8	−75.5	45.4	−23.3	67.9	−1.0
IM4 + 2HCl	−137.5	−161.9	−130.3	−154.5	−128.4	−152.6	−120.3	−143.1	−116.5	−138.7
Tsa6 + 2HCl	−9.3	−62.9	−1.7	−55.5	0.0	−53.9	5.5	−43.4	8.2	−38.7
TSb1	39.4	−3.7	37.9	−5.1	37.9	−5.1	89.3	47.3	110.8	69.2
Imb1 + HCl	−71.1	−83.2	−67.8	−80.0	−66.8	−79.0	−62.6	−72.6	−60.6	−69.7
TSb2 + HCl	44.6	4.9	48.8	8.6	49.7	9.2	50.6	14.8	51.1	17.2
Imb2 + 2HCl	−51.8	−59.6	−43.9	−51.7	−42.6	−50.4	−82.6	−88.7	−99.0	−104.5
TSb3 + 2HCl	6.0	−42.0	12.9	−35.9	14.2	−34.8	23.2	−17.8	27.2	−10.4
Imb3 + 3HCl	−115.6	−137.2	−104.0	−125.6	−101.8	−123.3	−137.9	−155.8	−152.6	−168.9
TSb4 + 3HCl	2.7	−42.0	15.2	−30.2	17.2	−28.4	−23.3	−61.3	−39.8	−74.7
TSb5 + HCl	−12.4	−76.9	−10.2	−74.0	−9.2	−72.4	44.8	−15.7	67.7	8.6
TSb6 + 2HCl	−12.6	−66.2	−4.8	−58.6	−3.0	−57.0	2.1	−47.6	4.6	−42.2
TDI + 4HCl	−97.7	−113.9	−81.4	−97.4	−78.8	−94.9	−160.0	−173.2	−193.5	−205.5

**Table 2:** Gas-phase thermochemical properties for intermediates of the phosgenation of the 2,4-TDA. The standard enthalpy of formation is calculated from G3MP2B3 enthalpies through group additivity (GA) role obtained by the means of online NIST tool [27] and through atomization scheme (AS). Standard molar enthalpy of formation (Δ_f,298.15K_*H*^0^(g)), standard molar entropy (*S*^0^(g)), and molar heat capacity (*C_v_*(g)) were obtained at 1 atm pressure at 298.15 K. Entropy and heat capacity values are obtained from scaled B3LYP/6-31G(d) frequencies.

Species	Δ_f,298.15K_*H*^0^(g) (kJ/mol) (GA)	Δ_f,298.15K_*H*^0^(g) (kJ/mol) (AS)	*S*^0^(g) (J/molK)	*C_v_*(g) (J/molK)
TDA	57.7	59.3	372.5	145.2
IMa1	n.a.	−135.5	454.3	183.2
IMa2	n.a.	−19.5	419.5	160.7
IMa3	n.a.	−211.2	496.6	197.3
IM4	n.a.	−329.7	531.7	220.5
Imb1	n.a.	−137.8	451.3	182.6
Imb2	n.a.	−20.6	412.8	159.7
Imb3	n.a.	−210.0	493.2	197.1
TDI	−97.1	−94.1	457.5	174.5

## Data Availability

The data presented in this study are available on request from the corresponding author. The data are not publicly available due to the policy of the University of Miskolc.
